# A 16-Year-Old Male With Frequent Nocturnal Events During Slow Wave Sleep on a Polysomnogram

**DOI:** 10.7759/cureus.18047

**Published:** 2021-09-17

**Authors:** Sameh S Morkous

**Affiliations:** 1 Pediatric Neurology, Lehigh Valley Health Network (LVHN) Lehigh Valley Reilly Children's Hospital, Allentown, USA

**Keywords:** pediatric sleep disorders, pediatric neurology, seizures, focal seizures, parasomnia

## Abstract

A 16-year-old male was referred by the primary care physician (PCP) for a second opinion. An initial evaluation in another sleep center suggested a working diagnosis of night terrors for the last two years. The child would wake up frequently screaming for few minutes before going back to sleep with no recollection of these events later. A video during the polysomnography (PSG) showed the patient having one of his typical events. He was eventually diagnosed with Sleep-related Hypermotor Epilepsy (SHE) seizures. This case highlights the importance of differentiating parasomnia and seizures, particularly for the sleep medicine providers that incorporate providers from different academic backgrounds. We will discuss the clinical challenges to make the distinction for the referring providers and demonstrate the importance of video-PSG to establish the diagnosis.

## Introduction

The differential diagnosis between Sleep-related Hypermotor Epilepsy (SHE) and parasomnias may be challenging, even for experts in epilepsy and sleep medicine, due to the potential similarities between these two sleep-related manifestations. Misdiagnosis is frequent in SHE patients because of the presence of behavioral patterns similar to those observed in non-rapid eye movement (NREM) parasomnias and rapid eye movement (REM) behavior disorders. Background activity on the electroencephalogram (EEG) can be normal in about half of SHE cases, creating additional challenges for the appropriate diagnosis. Interictal EEG is normal in about half of cases or may demonstrate rare epileptiform abnormalities enhanced by sleep deprivation and occurring mainly during sleep. The ictal scalp EEG may be normal or may only demonstrate movement artifacts. Epileptiform abnormalities, rhythmic slow activity, or diffuse background flattening over frontal areas are seen in 50-60% of cases. A large cohort study reported a diagnostic delay of 12.8±10.1 years in 53.7% of SHE cases, with parasomnias being the most frequent misdiagnosis (55.5%) [1]. It is essential to recognize the clinical cues that would raise concerns for an underlying seizure disorder like SHE to provide an early referral to the consultant.

## Case presentation

A 16-year-old male presented to the sleep lab for routine nocturnal polysomnography (PSG) ordered by the pediatrician followed by the sleep specialist consultation with a working diagnosis of frequent night terrors that were provided from another sleep center. These episodes began two years ago and were initially occurring once or twice weekly but were getting more frequently now occurring few times every night. The child would wake up screaming and shaking for few minutes before going back to sleep, with no recollection of these events. Epworth Sleepiness Scale (ESS) assessment was 8/24. He did snore with occasional reported respiratory pauses, raising concerns for underlying obstructive sleep apnea, for which the pediatrician, primarily, ordered the PSG. At bedtime (10 pm), he would fall asleep within 15 minutes; in the morning, he would wake up at 7:30- 8:00 am, with no daily naps. There was no other significant medical history and no family history of parasomnias or seizures.

Examination revealed patient weight at 73.3 kg; height, 165.5 cm. Z-scores of 0.79 (79%) and -1.25 (10%) were based on CDC 2-20 years weight-for-age, and stature-for-age data, respectively. Body mass index was 26.76 kg/ (m^2^) (Z score: 1.44, 93rd percentile). He had a Mallampati score of 1; tonsil size of 1+. The remainder of the physical examination was normal.

**Figure 1:**
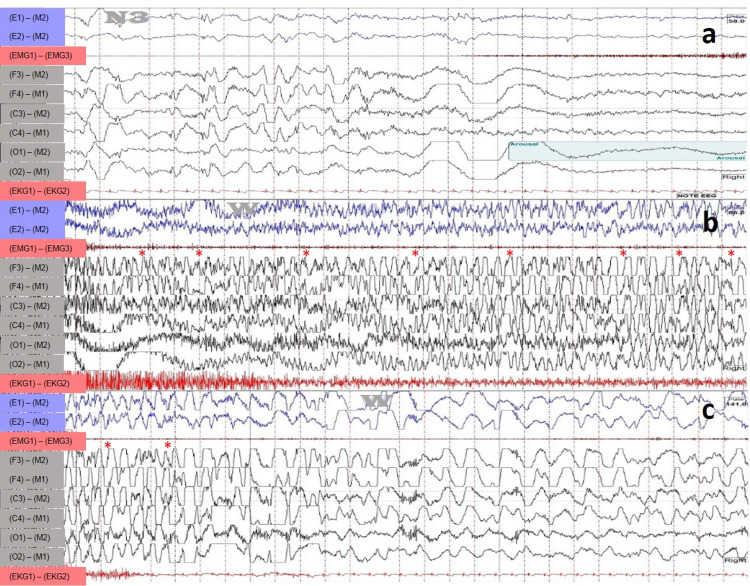
Frontal lobe seizure with an EEG 30 seconds window on the sleep study (a) The onset of event from N3; (b) Continuation; & (c) The end of the event Asterisks in (b) and (c) refer to individual epileptiform discharges. This event occurred at 6:01 am.

The PSG revealed total sleep time as 322.7 minutes; lights-off time, 10:15 pm; lights-on time, 6:12 am. The obstructive apnea-hypopnea index was 0.4 events/h, the lowest oxygen saturation was 94%, and the mean end-tidal carbon dioxide (EtCO2) was 40.4 mmHg. Periodic limb movement index was 1.0 events/h. However, during the PSG, the patient had three typical events at 3:05 am, 4:44 am and 6:01 am. During these events, the patient would wake up confused with the extension of his left arm, head rotation to the left, flexion of the right arm, and abduction of the right shoulder. Each episode lasts for 10-15 seconds, followed by falling back to sleep. The following changes were seen on the EEG in a 30 seconds window on the sleep study (Figure 1).

The diagnosis is SHE seizures, also known as nocturnal frontal lobe epilepsy (NFLE). Figure 1 above demonstrates a 30 seconds window of the EEG portion on the sleep study with (1a) onset of event from N3; (1b) continuation and (1c) end of the event. Asterisks in Figure (1b/1c) refer to the individual epileptiform discharges. The semiology with the “fencing posture” described above is a classic manifestation of seizures arising from the supplementary sensorimotor area of the frontal lobe and in the context of the EEG findings exhibited above will support the diagnosis. The most appropriate next step is to rule out any structural cerebral abnormalities that might cause the SHE, a form of focal epilepsy, and to start an antiepileptic medication (carbamazepine).

Follow up

**Figure 2:**
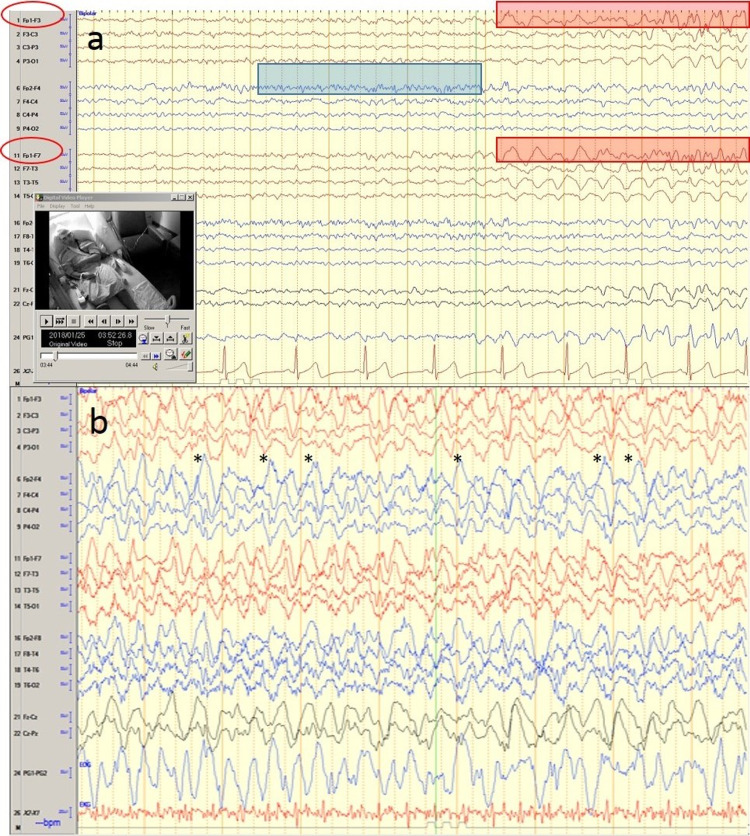
The 20-channel bipolar longitudinal electroencephalogram (EEG) montage of a 10-second window using the standard international 21 electrode positions in the 10–20 system (a) The onset of the patient’s typical seizure is shown by fast beta activity (blue highlight) at the right frontal region (FP2-F4 electrodes) that then evolved to the left frontal region (FP1-F3 electrodes/FP1-F7 electrodes) with rhythmic delta and theta activity (red highlighted areas). (b) This subsequently evolved over the next 5-10 seconds to the rapid rhythmic generalized epileptiform discharges shown (asterisks refer to the individual epileptiform discharges). Time base: 10mm /sec, Sensitivity: 7uV (microvolts) /mm. This event occurred at 3:52 am.

The patient was admitted to the hospital for further evaluation, where his brain MRI was found normal. He also had additional inpatient standard video EEG monitoring during which his seizures were demonstrated again (Figure 2). His seizures were heralded by fast beta activity at the right frontal region (FP2-F4 electrodes) that then evolved to the left frontal region (FP1-F3 electrodes/FP1-F7 electrodes) with rhythmic delta and theta activity. This subsequently evolved over the next 5-10 seconds to rapid rhythmic generalized epileptiform discharges (highlighted with asterisks). The patient was started on carbamazepine. The patient tolerated his medication well and reported no side effects on the three-month follow-up.

## Discussion

A PubMed advanced search for the search terms "NFLE" AND "adult case" yielded 23 articles, most of these articles were discussing the pathophysiology and/or the management aspects of SHE/NFLE. Yeh and Schenck reported two additional cases of sporadic (i.e. non-familial) NFLE and integrated these two cases within the first series of 10 cases of sporadic NFLE reported in Taiwanese patients where they reported that the cases corresponded closely to the previously reported sporadic and familial NFLE among Caucasian patients in Europe and North America [2]. There was a high rate of sustained anticonvulsant treatment efficacy, particularly with carbamazepine, oxcarbamazepine, and topiramate, and four of the 10 patients had hypermotor manifestations of their NFLE including one of the two newly reported cases and they discussed the newly published entity of SHE at the time [2]. A similar PubMed search in pediatrics for the search terms "NFLE" AND "pediatric case" yielded six articles discussing either semiology and/or management for SHE with a one case report describing NFLE in mucopolysaccharidosis where the long term video-EEG monitoring (LT-VEEGM) demonstrated sleep-related hypermotor seizures consistent with NFLE [3]. 

The differential diagnosis of paroxysmal events during sleep includes parasomnias, non-rapid eye movement (NREM) arousal disorders, psychogenic nonepileptic seizures, and dissociative disorders [4]. NREM arousal disorders include confusional arousal (CoA) episodes, typically occurring several times per month and characterized by disoriented behavior with a poor recall of events. Other NREM arousal disorders are sleepwalking associated with ambulation, and sleep terror that is distinguished by diaphoresis and lack of recall. It is rare for sleep terrors to emerge multiple times nightly for extended periods of time [4]. Rapid eye movement (REM) sleep behavior disorder was ruled out in our patient due to the preservation of the normal REM sleep atonia on the polysomnography. Diagnosis of non-epileptic seizures is challenging; although history and interview can correctly characterize up to 86% of patients. Video electroencephalography (video-EEG; the gold standard) is also normal [5]. Sleep-related dissociative disorders are characterized by dissociative episodes that occur near sleep-wake transitions, and unlike other parasomnias, they arise from well-established EEG wakefulness.

Sleep-related hypermotor epilepsy (SHE), also known as nocturnal frontal lobe epilepsy, is characterized by frequent, brief (<2 minutes) seizures with stereotyped motor patterns and abrupt onset and offset. The most common clinical expression consists of “hypermotor” events. SHE seizures occur predominantly during sleep but may also occur during wakefulness. They may be preceded by abrupt arousal, similar to our patient, or a distinct aura [6]. NREM parasomnia usually emerges from a slow sleep stage, typically within 2 hours of sleep onset, although this is not a major element for the differential diagnosis [6]. SHE seizures occur during any sleep stage but are common shortly after falling asleep [4], usually occur out of stage N2, and have been reported during slow-wave sleep, similar to our case [7]. Differentiating nocturnal seizures from NREM parasomnias can be challenging (Table 1 ).

**Table 1:** Semiological distinctions between NREM arousal disorders, NFLE, and RBD NREM - Non-rapid eye movement; REM - Rapid eye movement; RBD - REM Behavior Disorder; NFLE - Nocturnal Frontal lobe Epilepsy References [8,9]

Features	NREM arousal disorders: confusional arousal, sleep-walking, and sleep terrors	NFLE	RBD
Time of night	Children: early half of the night usually the first third of the night. Adults: early or late	Any time, often in clusters	Late
Sleep stage at the start	Children: stage N3. Adults: stage N2 or N3	NREM > Wake > REM	REM
Age of onset	Childhood	Childhood or Adolescence or adults	Adults > 50 years
Screams	Yes, in sleep terrors. No, in confusional arousals and sleep-walking where walking is typical in sleep-walking	Rare	Can occur, talking and yelling more common
Motor features	Variable, not highly stereotyped, no dystonic posture	Highly stereotyped, often hyperkinetic	The defining symptom of RBD is repeated episodes of sleep-related vocalization and/or complex motor behaviors during REM sleep, correlating with dream mentation.
Amnesia for the events and confusion upon awakening	Generally present	Generally present	Generally absent
Postictal confusion/amnesia	Frequent, long duration	Absent or short duration	Generally absent
Ictal EEG	Slow waves	Clear-cut epileptiform discharges < 10 %	The characteristic polysomnographic finding of RBD is REM sleep without atonia (RWA), which is an elevation of motor tone during REM sleep as measured by electromyography (EMG) in the chin and/or limb leads

While it is well known that frontal lobe epilepsy may manifest with focal seizures during sleep, nocturnal temporal lobe epilepsies (NTLE) have received less attention (Table 2). Recognizing this entity might be crucial as, within the temporal lobe epilepsy, the NTLE form seems to have a better surgical prognosis [10]. Standard PSG typically utilizes 4-6 channels of EEG, which is inadequate to evaluate focal epileptiform activity. The American Clinical Neurophysiology Society recommends extended EEG monitoring with ≥16 derivations in bipolar and referential montages and increasing the high pass filter to 70 Hz to evaluate [11] especially if seizures are suspected. Extended EEG montage and visualization of waveforms in a 10-second window are recommended [9].

**Table 2:** Semiological distinctions between temporal and frontal lobe seizures Reference [7]

Features	Temporal Lobe	Frontal Lobe
Seizure Characteristics		
Frequency	Less frequent	Frequent (often daily)
Duration	Longer	Brief
Manifestations		
Complex Posture	Less frequent	Frequent and prominent
Prominent Motor Activity	Rare	Common
Vocalization	Speech (non-dominant)	Loud non-speech (grunts, screams, or moans)
Post-ictal confusion/amnesia	Long duration	Short duration

SHE predominates in males (7:3), with onset usually during infancy and adolescence [12]. SHE is autosomal dominant or sporadic, and causes can also include acquired injuries and structural anomalies such as focal cortical dysplasia. In 1977, Pedley and Guilleminault described an unusual type of sleepwalking in six patients, all characterized by screaming, vocalization, and complex automatisms. The episodes ceased after specifically using phenytoin or carbamazepine treatment. Four of these patients demonstrated epileptiform abnormalities in their electroencephalograms (EEGs). Based on the EEG abnormalities and the favorable response to therapy, the authors concluded that these episodes are epileptic in nature although the EEG recordings of two of the attacks did not demonstrate apparent EEG abnormalities [13]. Subsequently, a few years later in 1981 , Lugaresi and Cirignotta reported additional cases with complex motor attacks recurring every night during slow-wave sleep, where the authors strongly argued about the epileptic versus non-epileptic nature of these episodes and they were ultimately considered to be a form of a rare motor disorders of sleep origin mainly because of the complex motor pattern that occurred only during sleep and the absence of definite EEG epileptiform abnormalities [13]. The term “hypnogenic paroxysmal dystonia” was used first, and then in 1981, the misleading term “Nocturnal Paroxysmal Dystonia (NPD)” was introduced by Lugaresi et al. [13]. Subsequently, in 1985 and 1987, Williamson et al. reported the similarity of the attacks to those in patients with frontal lobe epilepsy undergoing neurosurgical evaluation, and in 1990 Tinuper et al. documented epileptiform discharges in three patients which led to the disorder being renamed as NFLE [14]. In the next two decades, controversial issues highlighted the need for a more accurate nomenclature where a large video-polysomnographic study of 100 consecutive NFLE cases by Provini et al. (1999), highlighted the different intensities and durations of these sleep-related manifestations that can occur in a single patient and even in a single night [13]. The postulation that these episodes could represent a continuum of the same seizure disorder was established by further studies by Nobili et al. (2007), by using intracerebral EEG recordings techniques during the presurgical evaluation of the drug-resistant SHE [14] . Finally, in 2014, a Consensus Conference was held in Bologna, Italy where experts in the field agreed to change the nomenclature to SHE as the seizures may also arise from extra-frontal areas; and to put more stress, that though seizures can occur during sleep but also can take place both during the day and/or the night independent of the daytime; and eventually to better define the seizures semiology primarily as being hyperkinetic seizures. Experts further agreed that SHE should be considered a unique syndrome regardless of its underlying etiology or the brain area involved [13,14]. In many patients with SHE, the EEG is normal. The yield of surface EEG may be limited due to the difficulty in the detection of mesial or basal foci, and the patient may be misdiagnosed as having non-epileptic events. In addition, in patients with mesial frontal foci, the epileptiform discharges may be mislateralized ("paradoxical lateralization") [15]. The prolonged video-EEG, the best tool to assess seizure occurrence, is obligatory to characterize the abnormalities in patients undergoing presurgical investigation [4,6]

 The first medication of choice is usually carbamazepine [16], which controls the seizures in ~20% of the cases and reduces seizures by half in another 48% [12]. However, this therapy is ineffective in at least one-third of patients [6,12]. Other antiepileptic medications include valproic acid, gabapentin, lamotrigine, levetiracetam, oxcarbazepine, and topiramate [17]. Epilepsy surgery has been effective in some patients with drug-resistant SHE related to focal cortical dysplasia [5,8]. Vagus nerve stimulation might help but is inferior to resective epilepsy surgery [18].

## Conclusions

This case finely delineated the characteristic findings of SHE from the Sleep Medicine perspective, where this medical condition can be misdiagnosed as a form of parasomnia because the events can occur primarily during sleep. We also discussed the key features to support an epileptic etiology of the paroxysmal events and the essential cues to differentiate nocturnal seizures versus parasomnias. An astute clinician would recognize the characteristic features discussed in our case, in particular, “the fencing posture,” and also the salient clinical attributes that characterize SHE from parasomnia events primarily the nocturnal, stereotypical, hypermotor, repetitive and brief events; all of these features would be highly suggestive of medical conditions like SHE rather than night terrors or nightmares. Also, a perspicacious physician would consider an extended EEG monitoring when PSG is used if there are clinical concerns for seizures, especially SHE.

Recognizing all of this in patients by the providers is pivotal to be able to differentiate parasomnias from underlying seizures, especially in the Sleep Medicine field. It is crucial to provide an early referral for evaluation and management to avoid unnecessary delays in providing medical care.
